# Stability of SARS-CoV-2 in Biological Fluids of Animals

**DOI:** 10.3390/v15030761

**Published:** 2023-03-16

**Authors:** Taeyong Kwon, Natasha N. Gaudreault, Konner Cool, Chester D. McDowell, Igor Morozov, Juergen A. Richt

**Affiliations:** Department of Diagnostic Medicine and Pathobiology, College of Veterinary Medicine, Kansas State University, Manhattan, KS 66506, USA

**Keywords:** animal biological fluids, cat, SARS-CoV-2, sheep, stability, white-tailed deer

## Abstract

Since its first emergence in 2019, severe acute respiratory syndrome coronavirus 2 (SARS-CoV-2) has continued to evolve genetically, jump species barriers, and expand its host range. There is growing evidence of interspecies transmission including infection of domestic animals and widespread circulation in wildlife. However, knowledge of SARS-CoV-2 stability in animal biological fluids and their role in transmission is still limited as previous studies focused on human biological fluids. Therefore, this study aimed to determine the SARS-CoV-2 stability in biological fluids from three animal species, cats, sheep and white-tailed deer (WTD). Saliva, feces, 10% fecal suspensions, and urine of cats, sheep, and WTD were mixed with a known concentration of virus and incubated under indoor and three different climatic conditions. Our results show that the virus was stable for up to 1 day in the saliva of cats, sheep, and WTD regardless of the environmental conditions. The virus remained infectious for up to 6 days in feces and 15 days in fecal suspension of WTD, whereas the virus was rather unstable in cat and sheep feces and fecal suspensions. We found the longest survival of SARS-CoV-2 in the urine of cats, sheep, and WTD. Furthermore, side-by-side comparison with different SARS-CoV-2 strains showed that the Alpha, Delta, and Omicron variants of concern were less stable than the ancestral Wuhan-like strain in WTD fecal suspension. The results of our study provide valuable information for assessing the potential role of various animal biological fluids in SARS-CoV-2 transmission.

## 1. Introduction

Severe acute respiratory syndrome coronavirus 2 (SARS-CoV-2) is the etiological agent of coronavirus disease 2019 (COVID-19) and is responsible for the current pandemic. The emerging, zoonotic virus was first identified in December 2019 in China, and subsequently spread across the globe causing significant impact to public health and the global economy. SARS-CoV-2 is a member of genus *Betacoronavirus* in the family *Coronaviridae* of the order *Nidovirales* and causes a wide range of symptoms. The elderly and people with underlying medical conditions are at higher risk for severe illness. Coronaviruses are known to cross species barriers and have expansive host ranges, with bats being regarded in many cases as their natural reservoirs.

More than 20 years ago, a cousin of the current pandemic virus, SARS-CoV-1 caused the first SARS outbreak, with the first cases reported in 2002, in Guangdong province, China. SARS-CoV-1 has an approximately 10% case fatality rate among the 8086 cases reported so far [1]. An initial study suggested that SARS-CoV-1 originated in wild animals, including palm civets, but subsequent genetic evidence supported a SARS-CoV-1 origin in horseshoe bats (genus *Rhinolophus*) [2,3]. Middle East respiratory syndrome coronavirus (MERS-CoV) also causes a severe respiratory illness in humans and first emerged in 2012. Molecular and serological surveillance studies indicate that the interspecies transmission of MERS-CoV may have first occurred from bats to dromedary camels, from which the virus was ultimately transmitted to humans [4,5,6]. There is speculation that other human coronaviruses such as 229E, NL63, OC43, and HKU1, also have zoonotic origins and emerged from bats or rodents before their spillover into human populations through intermediate hosts, such as camelids and bovines [7,8,9,10,11,12]. Current knowledge supports the zoonotic origin of SARS-CoV-2 derived from bats through intermediate hosts, such as pangolins [13,14]. Since SARS-CoV-2 was introduced into the human population, reverse zoonotic events from humans to animals have often been reported in companion animals, including cats, dogs, hamsters, and ferrets; farmed mink; captive zoo animals; as well as free-ranging and captive white-tailed deer (WTD). Under experimental conditions, a variety of animal species have been shown to be susceptible to SARS-CoV-2 infection, and the virus can be transmitted to naïve contact animals in many cases [15].

To date, investigations into the stability of SARS-CoV-2 have been directed toward contaminated surfaces [16,17,18] and human biological fluids [19,20,21]. Although human–human transmission primarily maintains the circulation of SARS-CoV-2, several lines of evidence have shown that secondary zoonotic transmission of SARS-CoV-2 has occurred from farmed mink [22,23], cats [24], WTD [25], or pet hamsters [26] to humans. Widespread and ongoing circulation of SARS-CoV-2 in wildlife have been reported [27,28,29], which may contribute to the emergence of novel variants and the re-introduction of these variant viruses into humans after long-term circulation and evolution in these animal settings.

The aim of this study was to evaluate the potential role of animal biological fluids in SARS-CoV-2 transmission. We added known quantities of different SARS-CoV-2 strains, including variants of concern (VOCs), to saliva, feces, 10% fecal suspensions, and urine from three animal species: cat, sheep, and WTD. The samples were then incubated under various environmental conditions and tested for the presence of infectious virus to determine virus stability and calculate the respective half-life values. We found that SARS-CoV-2 is stable in feces of WTD and in urine of all the animals investigated, but was less stable in saliva.

## 2. Materials and Methods

Saliva, feces, and urine from cats, sheep, and WTD were collected from our previous SARS-CoV-2 challenge studies [30,31,32,33] and stored at −20 or −80 °C before use (Table 1). A 10% fecal suspension (*w/v*) was prepared in phosphate buffered saline. A total of 50 μL of each biological fluid was inoculated onto Vero E6 cells or Vero-TMPRSS2 cells; all biological materials were confirmed negative for SARS-CoV-2. The SARS-CoV-2 USA-WA/2020 strain (herein WA-1) was obtained from BEI resources (BEI catalog number: NR-52281) and was propagated in Vero E6 cells in Dulbecco’s Modified Eagle Medium supplemented in 5% fetal bovine serum and 1% antibiotic-antimycotic solution (herein as virus growth medium). The virus was sequenced using Illumina NextSeq and the consensus sequence was determined to be identical to the reference sequence that was deposited in GISAID (Supplementary Table S1). However, a four amino acids insertion in the spike glycoprotein, KLRS (between amino acid positions 215–216 of the reference sequence), was found at a frequency of 44%. The virus stock was diluted to a concentration of 10^7^ TCID_50_/mL in virus growth medium. A total of 5 μL of 10^7^ TCID_50_/mL of SARS-CoV-2 (equivalent to 5 × 10^4^ TCID_50_) was mixed with 45 μL of each biological fluid or 0.1 to 0.2 g of feces in a sealed 2 mL tube because SARS-CoV-2-infected humans and animals shed up to 10^5^ infectious units per mL under experimental conditions. The same amount of virus was mixed with 45 μL of virus growth medium in a 2 mL tube as the positive control. The tubes were incubated in a chamber that has the capability to maintain a constant temperature and relative humidity (RH). The chamber was operated under indoor conditions (21 °C/60% RH) and three other conditions to simulate seasonal differences: 25 °C/70% RH for summer, 13 °C/66% RH for spring/fall, and 5 °C/75% RH for winter as described previously [18,19]. There were three replicates of each sample. Samples were removed from the chamber to determine the presence of infectious virus at 1 and 7 h post-contamination (hpc), and at 1, 2, and 3 days post-contamination (dpc) for indoor and summer conditions. For spring/fall conditions, 1 hpc and 1, 3, 5, and 7 dpc were used, as was 1 hpc and 1, 3, 6, 10, 15, and 21 dpc for winter conditions. After removal from the chamber, a total of 1.95 mL of virus growth medium was added to the tube and the tube vortexed thoroughly for 10 s. The supernatant was filtered through a 0.45 μm syringe filter to prevent bacterial contamination. Subsequently, 10-fold serial dilutions were prepared and inoculated onto Vero E6 cells. Cytopathic effects were determined at 4 days post-inoculation, and the virus titer was calculated using the Reed–Muench method.

To further assess the stability of SARS-CoV-2 variants of concern (VOCs) in fecal suspensions of WTD, the following VOC viruses were obtained from BEI (NR-54000; Alpha VOC) or Viviana Simon (Mount Sinai Pathogen Surveillance program; Delta and Omicron VOCs) via Michael Schotsaert (Icahn School of Medicine at Mount Sinai), and were grown in Vero-TMPRSS2 cells: isolate hCoV-19/England/204820464/2020 (NR-54000) for Alpha VOC, isolate hCoV-19/USA/NYMSHSPSP-PV29995/2021 for Delta VOC, and isolate hCoV-19/USA/NY-MSHSPSP-PV44476/2021 for Omicron VOC. The sequences of each virus stock were obtained by sequencing on the Illumina NextSeq or Miseq platform and all three strains were 100% homologous to the reference sequences that are deposited in GISAID [34]. The WA-1 strain was also grown in Vero-TMPRSS2 cells in order to prepare all virus inocula under identical condition; next generation sequencing revealed that it had 100% nucleotide identity to the reference sequence deposited in GISAID except for a synonymous mutation at position 1912 (C/T) in the nsp2 region. The virus stocks of the three VOCs and WA-1 were diluted in virus growth medium to a concentration of 6.2 × 10^6^ TCID_50_/mL. A total of 5 μL of each virus (equivalent to 3.1 × 10^4^ TCID_50_/mL) was mixed with 10% fecal suspension of WTD and incubated under winter conditions since the positivity rate of SARS-CoV-2 in WTD peaks in winter [29]. At 2 hpc and 1, 3, 6, and 10 dpc, the samples were processed as described above, and titrated on Vero-TMPRSS2 cells to identify the presence of infectious virus.

The virus decay rate of SARS-CoV-2 was estimated if the respective samples were virus positive for at least two time points. The virus titers from the first (i.e., 1 or 2 hpc) to the last positive time points were log-transformed, and a simple linear regression was estimated in Prism 9 (GraphPad, San Diego, CA, USA). The half-life value was calculated as −log_10_ (2)/slope. One-way analysis of variance (ANOVA) and subsequent Tukey’s multiple pairwise comparisons were performed to determine whether there was statistical evidence that the seasonal pattern of virus decay or differential virus stability of the VOCs were significantly different.

## 3. Results

**Figure 1:**
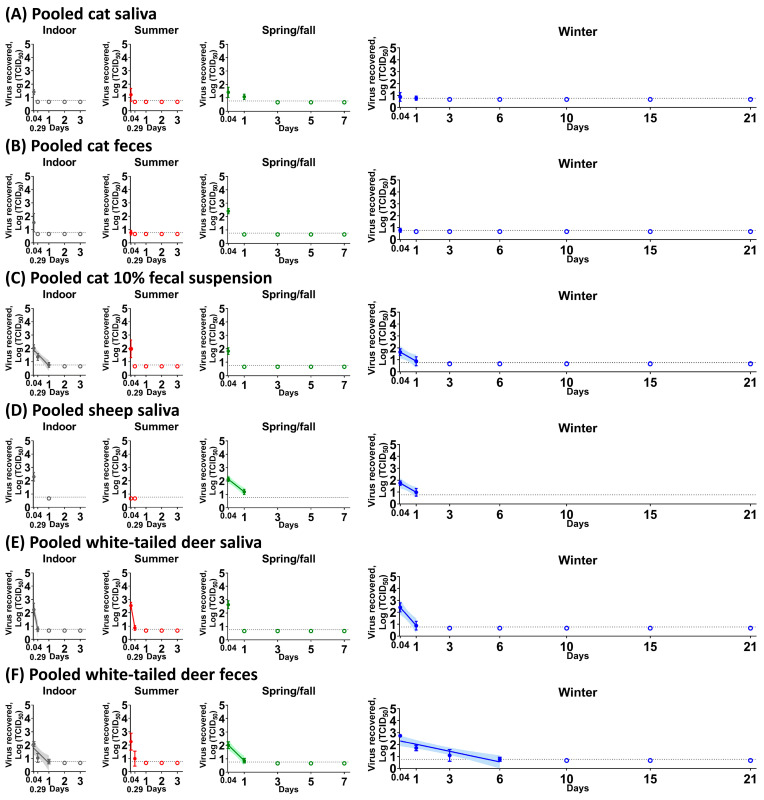
SARS-CoV-2 stability in pooled cat saliva (**A**), pooled cat feces (**B**), pooled cat 10% fecal suspension (**C**), pooled sheep saliva (**D**), pooled white-tailed deer saliva (**E**), and pooled white-tailed deer feces (**F**). Each biological fluid was spiked with 5 × 10^4^ TCID_50_ of SARS-CoV-2 and incubated under indoor (gray), summer (red), spring/fall (green), and winter (blue) conditions. At each time point, the virus was recovered and titrated on Vero E6 cells. The simple linear regression was estimated when the infectious virus was at least present at two different time points. The virus titer is represented as the mean and standard deviation of log transformed TCID_50_ titers (colored circle), whereas empty colored circles represent negatives in triplicate. Colored lines and their shaded areas represent a best-fit line and 95% confidence interval of the simple linear regression. On the x-axis, 0.04 and 0.29 days are equal to 1 and 7 h, respectively. Due to insufficient volume of pooled sheep saliva (**D**), the virus stability was observed at two time points. The best-fit line was not shown in pooled cat saliva under spring/fall and winter conditions (**A**), and pooled white-tailed deer feces under summer conditions (**F**) because the slope of simple linear regression was not significantly different than zero.

**Figure 2:**
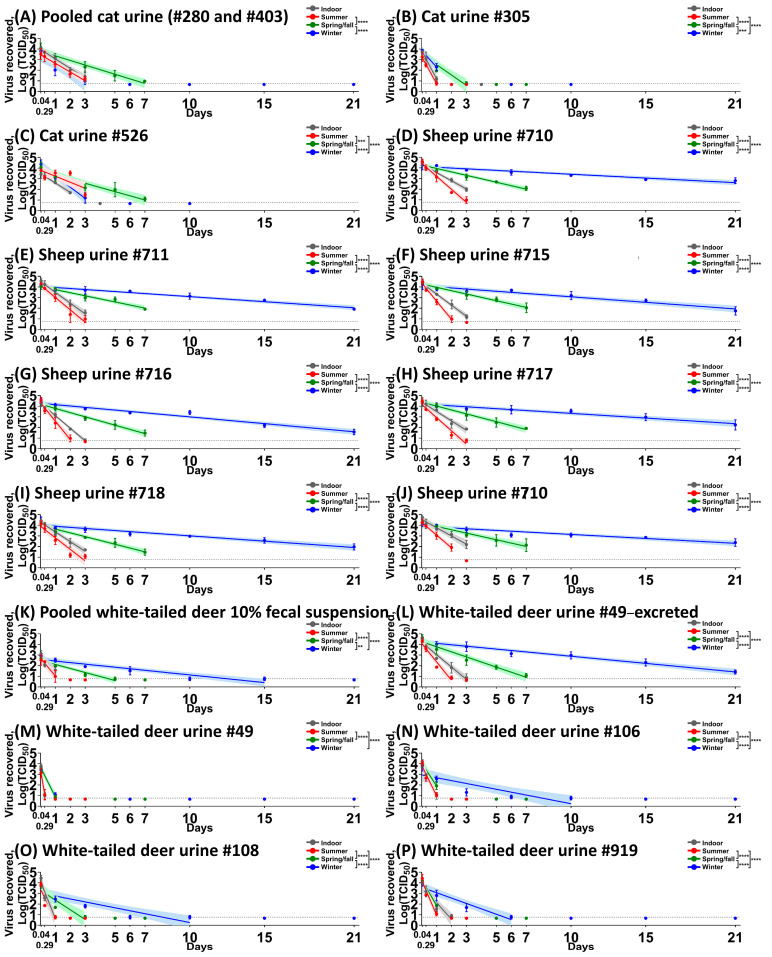
SARS-CoV-2 stability in cat urine (**A**–**C**), sheep urine (**D**–**J**), white-tailed deer 10% fecal suspension (**K**), and white-tailed deer urine (**L**–**P**). Each biological fluid was spiked with 5 × 10^4^ TCID_50_ of SARS-CoV-2 and incubated under indoor (gray), summer (red), spring/fall (green), and winter (blue) conditions. At each time point, virus was recovered and titrated on Vero E6 cells. The simple linear regression was estimated to calculate the half-life value and compare the values between three climate conditions using ANOVA and subsequent post hoc Tukey’s test. The virus titer is represented as the mean and standard deviation of log-transformed TCID_50_ titers (colored circles); colored lines and their shaded areas represent a best-fit line and 95% confidence interval of the simple linear regression. Adjusted *p*-values for significance are marked: ** (*p* < 0.01), *** (*p* < 0.001), and **** (*p* < 0.0001). On the x-axis, 0.04 and 0.29 days are equal to 1 and 7 h, respectively. Time points were 1 and 7 h, and 1, 2, and 4 days for cat urine #305 and cat urine #526 under indoor conditions.

In all experiments, except for the side-by-side comparison in 10% WTD fecal suspension, the starting virus load that was added to the different biological samples was 5 × 10^4^ TCID_50_. Following virus incubation with cat biological fluids, low levels of infectious virus, 10^0.767^ to 10^1.633^ TCID_50_, was isolated for up to 1 dpc in pooled cat saliva regardless of the environmental conditions (Figure 1A). In pooled cat feces, the virus titer ranged from 10^0.767^ to 10^2.412^ TCID_50_ at 1 hpc, and infectious virus was undetectable by 7 hpc (Figure 1B). In the pooled cat 10% fecal suspension (Figure 1C), the virus was able to survive for up to 1 day under indoor and winter conditions, with half-life values of 5.99 h and 9.16 h, respectively (Table 1). In contrast, we observed longer virus survival in the cat urine. Under spring/fall conditions, the virus was stable for up to 3 days or 7 days (Figure 2A–C) in pooled as well as individual #305 and #526 cat urine samples (Table 2). Interestingly, half-life values under winter conditions were significantly shorter than under spring/fall conditions in all three cat urine samples.

Virus incubation with sheep biological fluids revealed that in pooled sheep saliva (Figure 1D), a titer of 10^2.301^ TCID_50_ of SARS-CoV-2 was recovered at 1 hpc under indoor conditions, whereas no virus was isolated under summer conditions. The virus was stable for up to 1 dpc in pooled sheep saliva under spring/fall and winter conditions (Figure 1D), with half-life values of 7.34 and 9.04 h, respectively (Table 2). However, no infectious virus was recovered from pooled sheep feces or 10% fecal suspension at any of the time points. The virus survived in urine obtained from individual animals for up to 3 days under indoor, 2 or 3 days under summer, for 7 days under spring/fall, and 21 days under winter conditions (Figure 2D–J). For all sheep urine samples, the half-life under winter conditions was significantly longer than the half-life under summer and spring/fall conditions, with the virus being also significantly more stable under spring/fall conditions compared to summer conditions.

Virus incubation with WTD biological fluids revealed that the virus survived for up to 1 dpc in pooled saliva of WTD with half-life values of 1.23 h for indoor conditions, 1.08 h for summer conditions, and 4.52 h for winter conditions (Figure 1E and Table 2). The virus was very stable for up to 6 dpc in pooled WTD feces, with a half-life of 6.28 h under indoor conditions, 6 h under spring/fall conditions, and 24.44 h under winter conditions (Figure 1F and Table 2). This finding stands in clear contrast to results with feces from cats, sheep, and humans [19]. In addition, SARS-CoV-2 was stable in pooled deer feces for up to 7 hpc even under summer conditions (Figure 1F). We found longer virus survival in pooled 10% fecal suspension than in pooled feces of WTD where it survived up to 15 dpc compared to 6 days, respectively, under winter conditions (Figure 2K). These data reveal a seasonal pattern for virus stability in pooled WTD 10% fecal suspensions and feces, with the virus being least stable under summer conditions (Figure 1F and Figure 2K).

We next tested the stability of SARS-CoV-2 in WTD urine. Two urine samples from the same donor (#49), and three from other individual donors were used. In excreted urine that was collected from WTD #49, the virus was stable for up to 21 dpc and exhibited a seasonal stability pattern (Figure 2L). In contrast, in urine of WTD #49 that was collected directly from the bladder at necropsy, the virus was stable only for up to 7 hpc under indoor and summer conditions and 1 dpc under spring/fall and winter conditions, indicating differential virus decay rates in urine originating from the same donor (Figure 2L,M) collected using different methods. In the other three WTD urine samples that were collected directly from the bladder of individual deer at necropsy, the half-lives were shorter than those that were observed in the excreted urine of deer #49 but higher than those in the urine of deer #49 collected directly from the bladder, indicating a donor-dependent decay rate for WTD urine (Figure 2N–P).

**Figure 3:**
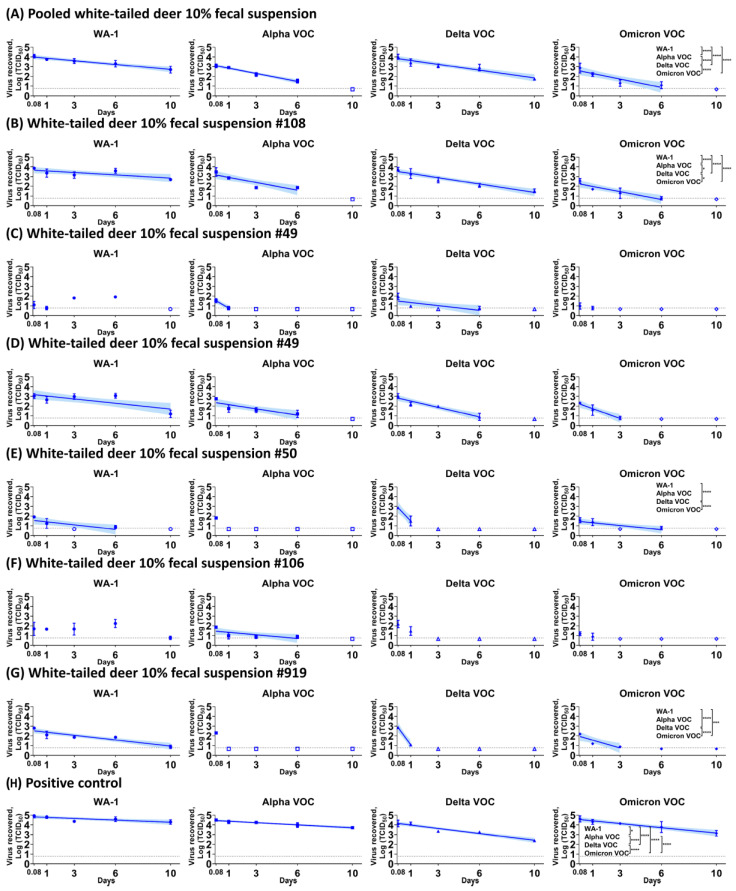
Stability of SARS-CoV-2 WA-1 strain and variants of concern in white-tailed deer 10% fecal suspension. A total of 3.1 × 10^4^ TCID_50_ of WA-1, Alpha, Delta, and Omicron VOCs were mixed with fecal suspensions obtained from different white-tailed deer (**A**–**G**) or medium alone (**H**) as a positive control and incubated under winter conditions. At each time point, virus was recovered and titrated on Vero-TMPRSS2 cells. The simple linear regression was estimated to calculate the half-life value and compare the values among strains using ANOVA and subsequent post hoc Tukey’s test. The virus titer is represented as the mean and standard deviation of log-transformed TCID_50_, and the colored line and their shaded areas represent a best-fit line and 95% confidence interval of the simple linear regression. Adjusted *p*-values for significance are marked: * (*p* < 0.05), *** (*p* < 0.001), and **** (*p* < 0.0001). On the x-axis, 0.08 is equal to 2 h. Pooled feces were collected on the animal room floor and mixed in PBS to prepare pooled 10% fecal suspension (**A**). Feces was collected from the anus of individual deer at 4 (**B**), 14 (**C**), and 18 (**D**–**G**) days post-challenge and mixed in PBS to prepare pooled 10% fecal suspension.

Lastly, we evaluated the stability of different VOCs in 10% fecal suspensions of several WTD under winter conditions. The WA-1 strain was significantly more stable than the Alpha, Delta, and Omicron VOCs under winter conditions (Figure 3 and Table 3). Although significant differences between the three VOCs were detected in each sample, we were unable to identify a consistent pattern of differences of stability for the VOCs in 10% fecal suspensions of WTD.

## 4. Discussion

The natural propensity of coronaviruses to cross species barriers can result in their (re-)emergence in humans and new animal species. Some coronaviruses can cause severe disease in humans, including SARS-CoV, MERS-CoV, and SARS-CoV-2. All of these viruses most likely originated from bats and were presumably introduced into human populations through different intermediate hosts. There are two porcine alphacoronaviruses, porcine epidemic diarrhea virus (PEDV) and swine acute diarrhea syndrome coronavirus (SADS-CoV), that cause severe acute gastroenteritis in neonatal piglets and are genetically closely related to bat coronaviruses, supporting the hypothesis that these viruses also emerged from bats [35,36,37,38]. Phylogenetic analyses have shown that a recently emerged porcine deltacoronavirus (PDCoV) shares a common ancestor with the sparrow coronavirus HKU17, which can experimentally infect chicks, turkey poults, and even gnotobiotic calves, supporting the hypothesis of an avian origin of PDCoV and its broad host range [39,40,41]. The potential of SARS-CoV-2 to be transmitted between animals and humans and vice versa has been highlighted by natural and experimental infections of a wide range of animal species [15]. This is supported by active and passive surveillance which reported the detection of SARS-CoV-2 RNA and antibodies in a multitude of animal species (www.oie.int (accessed on 9 February 2023)).

Cats are regarded as one of the important animal hosts due to their close contact with humans. Several experimental studies have demonstrated the susceptibility of domestic cats to SARS-CoV-2 infection. In experimental settings, viral RNA was detected in nasal swabs from days 1 to 13, oropharyngeal swabs from days 1 to 10, and rectal swabs from days 3 to 14 [30,42,43]. Infectious virus was isolated from the nasal swabs from days 1 to 7 and from oropharyngeal swabs from days 1 to 4, but no viable virus was isolated from the rectal swabs [44,45]. These experimental studies also showed cat–cat transmission of SARS-CoV-2 via close contact [30,44,45] and respiratory droplets [42]. Furthermore, natural transmission to cats in households with COVID-19 patients and from cats to people has been reported [15, EID citation], which suggests that cats may play a role in SARS-CoV-2 ecology and may constitute a virus reservoir. The present study adds to our knowledge of the potential risk of cat secretions and excretions in the indirect transmission of SARS-CoV-2. While it is less likely that the virus spreads indirectly through cat saliva and feces due to its short half-life in these biological samples, cat urine might serve as a source of infection under indoor conditions as the virus was detectable for up to 4 days.

Sheep are susceptible to infection by several coronaviruses. Bovine coronavirus and bovine-like coronavirus have been identified in the feces and intestinal content of healthy and diarrheic sheep, but coronavirus infections are considered low in prevalence with little to no impact on sheep health or production [46,47]. Studies that were conducted in Sweden and Ghana showed that 19.3% and 25.8%, respectively, of sheep sera were seropositive for bovine coronavirus antibodies [48,49]. More importantly, a growing body of evidence shows that sheep are susceptible to MERS-CoV infection. A serological surveillance study in Egypt revealed the presence of neutralizing antibody in sheep serum [50]. Subsequently, two experimental studies were performed to identify the susceptibility of sheep to MERS-CoV infection. In one study, low levels of infectious virus were isolated from the nasal swabs of two out of three experimentally infected sheep, and one sheep developed low-titer neutralizing antibody at day 14 [51]. Another study showed low-level viral RNA in nasal swabs on day 1 after infection, viral antigen in limited areas of the respiratory epithelium on day 2, but no seroconversion in 14 experimentally infected sheep [52]. These results suggest marginal susceptibility of sheep to MERS-CoV infection under experimental conditions. Interestingly, a recent study detected neutralizing antibodies against MERS-CoV in 35 of 63 (55.6%) sheep sera in close contact with camels in Senegal [53]. Likewise, recent studies demonstrated limited susceptibility of sheep to SARS-CoV-2 under experimental conditions [33,54]. It is possible that the close contact of humans to SARS-CoV-2 susceptible animals, such as domestic and feral cats or WTD on farms, live animal markets, and state/county fairs, could lead to accidental spillover of SARS-CoV-2 to sheep. In this context, contaminated urine from SARS-CoV-2 infected sheep could potentially pose a risk of zoonotic transmission to humans s since the virus was stable in sheep urine for several days under indoor and summer conditions and up to 21 days under winter conditions. Importantly, cleaning contaminated areas such as holding pens with water could generate aerosols and splash droplets, which could come in contact with the nasal and oral mucosa of humans and susceptible animals.

The susceptibility of WTD to SARS-CoV-2 was initially predicted in silico by the finding of conserved ACE2 binding residues similar to human ACE2 and the high propensity for binding to the SARS-CoV-2 RBD [55]. Subsequently, several experimental studies confirmed the SARS-CoV-2 susceptibility of WTD and virus transmission from infected to naïve deer [31,53,54,55,56,57]. In fawns, viral RNA shedding was found from 1 to 21 days post infection in nasal swabs, and from 1 to 7 days in rectal swabs [56]. Interestingly, infectious virus was isolated from 2 to 7 days post infection/post contact in nasal swabs of both inoculated and contact animals, and the virus titers ranged from 10^2^ to 10^4.8^ TCID_50_/mL. Furthermore, infected fawns shed infectious virus in feces with titers exceeding 10^5^ TCID_50_/mL. Our recent study in 2-year-old adult WTD demonstrated shedding of viral RNA for up to 10 days post infection and infectious virus for up to 5 days in nasal, oral, and rectal swabs, which was sufficient for efficient virus transmission to contact animals [31]. Recent surveillance studies have identified widespread SARS-CoV-2 infections in both free-living and captive WTD [27,28,29]. Importantly, genomic analyses of two independent studies that were conducted in Ohio [28] and Iowa [29] showed that multiple lineages of SARS-CoV-2 circulated in deer populations, and that the strains that were detected in deer formed several distinct clusters in phylogenetic trees, providing evidence of multiple spillover events from humans to WTD and subsequent deer–deer transmission. In fact, surveillance has identified spillover events of Alpha, Delta, and Omicron VOCs to WTD populations [58,59,60]. However, no study so far has addressed how SARS-CoV-2 is introduced into WTD populations. Here, we report that SARS-CoV-2 is stable only for up to 1 day in saliva, which may suggest that saliva is not a likely source of indirect transmission in WTD. In contrast, the virus survived under winter conditions for up to 6 days in feces and up to 15 days in 10% fecal suspensions.. In addition, SARS-CoV-2 is relatively stable in urine as evidenced in urine samples from different WTD. It is worthwhile noting that we obtained variable stability of SARS-CoV-2 in fecal suspensions from different individual deer and longer survival of the ancestral Wuhan-like lineage A strain when compared to the Alpha, Delta, and Omicron VOCs in fecal suspension. It is known that bucks are generally solitary while females form small family groups with their fawns during the spring and summer; however, in the fall and winter, individuals and family groups mingle to form larger groups [61,62]. Interactions within the group appear critical for direct deer–deer transmission once the virus is introduced into a deer population. In addition, it seems plausible that indirect transmission within and/or between groups might occur by grazing or around feeders in areas where infectious virus survives in feces and urine. It is also well-documented that feeding of deer carcasses resulted in deer–dog transmission of *Neospora caninum* [63] and deer–dog–human transmission of the Q fever agent, *Coxiella burnetii* [64]. It cannot be ruled out that SARS-CoV-2 might be transmitted to susceptible animal species through physical contact or consumption of deer carcasses or feces. In addition, there is a potential risk of infection to deer hunters when they handle the carcasses of infected animals.

There is no clear evidence of virus shedding in urine from infected cats, sheep, or WTD. One of the biggest obstacles is to collect clean urine from animals without environmental contamination. For this reason, experimental studies have investigated the presence of viruses in urine that was collected directly from the bladder at necropsy, which only gives limited information of virus presence at time of necropsy. For example, no viral RNA was detected in urine that was collected directly from the bladder of cats on 4, 7, and 21 days, sheep on 4, 8, and 21 days, or WTD on 4 and 18 days after infection [30,31,33], but there is a lack of knowledge for other time points. However, virus shedding via urine and its role in transmission should not be underestimated, since infectious virus has been detected in urine from humans and ferrets [65,66]. Here, we also tested the stability of different SARS-CoV-2 strains including VOCs in 10% fecal suspension of white-tailed deer. Continuous evolution of SARS-CoV-2 has led to the emergence of VOCs and subvariants, such as Omicron BA.5, which may exhibit different phenotypes including virus environmental stability. Vero E6 and Vero-TMPRSS2 cells were used for preparation of virus stocks and titration of samples since they have been widely used for SARS-CoV-2 research. However, recent studies have identified different routes of the Omicron VOC for entry into cells; thus, it cannot be excluded that a different virus entry mechanism may potentially affect our results [67]. Lastly, nasal excretions were not tested in this study due to the technical difficulty in collecting them from animals. Testing the stability in nasal excretions would provide valuable insight in its role in SARS-CoV-2 transmission since the virus is mainly excreted from the respiratory tract.

Overall, our study has implications for assessing the stability of SARS-CoV-2 in biological fluids from susceptible animals. The virus survived for up to 1 day post contamination in cat, sheep, and WTD saliva. The virus was isolated for up to 6 days in feces and up to 15 days in fecal suspensions of WTD, whereas it was rather unstable in cat and sheep feces and their fecal suspensions. The virus seems most stable in urine from cats, sheep, and WTD, where it exhibited a seasonal pattern of stability. We also found that virus survival in urine from cats and WTD as well as in fecal suspensions of WTD was donor-dependent. Furthermore, SARS-CoV-2 VOCs were less stable than the ancestral Wuhan-like strain in fecal suspensions of WTD. Although the exact role of biological secretions and excretions in SARS-CoV-2 transmission still remains unclear, our findings provide new insight into their potential role in intra- and inter-species transmission of SARS-CoV-2 and can contribute to the development of countermeasures against SARS-CoV-2 with the goal to mitigate zoonotic spillover events and circulation in animal populations.

## Figures and Tables

**Table 1:** List of biological fluids from cats, sheep, and white-tailed deer that were used in this study.

Species	Type	Pooled or Animal ID	Collection Method	Reference
Cat	Saliva	Pooled	Collection under sedation	[30,32]
	Feces	Pooled	Collection on floor	
	10% fecal suspension	Pooled	Collection on floor	
	Urine	Pooled from mock animals	Direct collection from bladder at necropsy	[30]
	Urine	#305	Direct collection from bladder at necropsy	[32]
	Urine	#526	Direct collection from bladder at necropsy	
Sheep	Saliva	Pooled	Collection under sedation	[33]
	Feces	Pooled	Collection on floor	
	10% fecal suspension	Pooled	Collection on floor	
	Urine	#710	Direct collection from bladder at necropsy	
	Urine	#711	Direct collection from bladder at necropsy	
	Urine	#715	Direct collection from bladder at necropsy	
	Urine	#716	Direct collection from bladder at necropsy	
	Urine	#717	Direct collection from bladder at necropsy	
	Urine	#718	Direct collection from bladder at necropsy	
	Urine	#719	Direct collection from bladder at necropsy	
White-tailed deer	Saliva	Pooled	Collection under sedation	[31]
	Feces	Pooled	Collection on floor	
	10% fecal suspension	Pooled	Collection on floor	
	10% fecal suspension	#49, 14 dpi	Direct collection from anus	
	10% fecal suspension	#49, 18 dpi	Direct collection from anus	
	10% fecal suspension	#50, 18 dpi	Direct collection from anus	
	10% fecal suspension	#106, 18 dpi	Direct collection from anus	
	10% fecal suspension	#108, 4 dpi	Direct collection from anus	
	10% fecal suspension	#919, 18 dpi	Direct collection from anus	
	Urine	#49	Collection from urination	
	Urine	#49	Direct collection from bladder at necropsy	
	Urine	#106	Direct collection from bladder at necropsy	
	Urine	#108	Direct collection from bladder at necropsy	
	Urine	#919	Direct collection from bladder at necropsy	

dpi = days post infection; #: Animal ID number.

**Table 2:** The half-life values of SARS-CoV-2 in biological fluids from cat, sheep, and white-tailed deer under indoor and three different climatic conditions.

Species	Type	Indoor		Summer		Spring/Fall		Winter	
		Half-Life (Hours)	95% C.I. ^1^ (Hours)	Half-Life (Hours)	95% C.I. ^1^ (Hours)	Half-Life (Hours)	95% C.I. ^1^ (Hours)	Half-Life (Hours)	95% C.I. ^1^ (Hours)
Cat	Pooled 10% fecal suspension	5.99	4.15, 10.79	N/D ^2^		N/D ^2^		9.16	4.61, 793.02
	Pooled urine (#280 and #403)	8.02	6.75, 9.86	8.84	7.24, 11.34	16.67	13.69, 21.31	8.14	5.78, 13.79
	Urine #526	8.10	6.7, 10.24	12.74	8.24, 28.06	18.38	14.51, 25.11	6.89	5.61, 8.92
	Urine #305	2.71	2.16, 3.67	2.84	2.36, 3.55	7.42	5.6, 10.96	4.62	3.31, 7.67
Sheep	Pooled saliva	N/D ^2^		N/D ^2^		7.34	5.13, 12.91	9.04	5.09, 40.6
	Urine #710	9.33	8.17, 10.9	5.90	5.19, 6.83	22.30	19.12, 26.74	99.95	82.68, 126.33
	Urine #711	7.12	6.12, 8.51	6.19	5.19, 7.66	24.95	21, 30.73	75.85	65.88, 89.38
	Urine #715	6.92	6.16, 7.9	4.21	3.72, 4.86	23.00	19.26, 28.57	69.54	58.1, 86.58
	Urine #716	6.10	5.65, 6.64	4.16	3.48, 5.16	18.32	15.92, 21.55	56.66	49.87, 65.59
	Urine #717	8.16	6.95, 9.87	5.80	5.05, 6.82	19.67	16.55, 24.2	81.83	66.83, 105.56
	Urine #718	7.88	6.73, 9.52	6.64	5.47, 8.43	20.25	17.25, 24.52	72.59	61.07, 89.49
	Urine #719	9.29	7.33, 12.68	6.04	5.1, 7.41	23.07	18.55, 30.52	95.12	78.01, 121.78
White-tailed deer	Pooled saliva	1.23	0.79, 2.75	1.08	0.84, 1.53	N/D ^2^		4.52	2.91, 10.15
	Pooled feces	6.28	3.66, 22.25	Not significant ^3^		6.00	4.2, 10.47	24.44	17.07, 42.95
	Pooled 10% fecal suspension	5.08	3.27, 11.39	4.31	2.9, 8.38	19.12	15.77, 24.26	49.94	40.64, 64.78
	Urine #49-excreted	6.22	5.35, 7.41	4.15	3.48, 5.15	15.11	12.66, 18.73	53.76	46.97, 62.86
	Urine #49-at necropsy	0.65	0.54, 0.84	0.89	0.59, 1.84	2.56	2.17, 3.13	2.97	2.51, 3.65
	Urine #106-at necropsy	2.24	1.92, 2.69	2.51	1.99, 3.42	3.37	2.49, 5.2	26.62	18.89, 45.12
	Urine #108-at necropsy	2.04	1.6, 2.83	2.55	1.79, 4.47	7.91	5.62, 13.36	26.09	19.53, 39.29
	Urine #919-at necropsy	4.58	3.59, 6.32	2.22	1.77, 2.96	2.97	2.59, 3.49	14.27	11.23, 19.57
Medium (positive control)		15.59	11.76, 23.11	10.56	8.21, 14.79	59.49	44.01, 91.73	156.55	106.26, 297.47

^1^ 95% C.I.: 95% confidence interval. ^2^ N/D: Not determined because infectious virus was present only at first time point. ^3^ The slope of simple regression model was not significantly different than zero. #: Animal ID number.

**Table 3:** The half-life values of SARS-CoV-2 WA-1 strain and different variants of concern in 10% fecal suspension of white-tailed deer under winter conditions.

Deer ID and Collection Time	WA-1		Alpha		Delta		Omicron	
	Half-Life (Hours)	95% C.I. ^1^ (Hours)	Half-Life (Hours)	95% C.I. ^1^ (Hours)	Half-Life (Hours)	95% C.I. ^1^ (Hours)	Half-Life (Hours)	95% C.I. ^1^ (Hours)
Pooled	56.62	43.7, 80.39	26.62	22.57, 32.44	36.23	28.98, 48.33	25.30	17.44, 46.15
#108, 4 dpc	88.78	52.21, 296.29	27.32	18.8, 49.99	33.40	27.1, 43.51	27.10	19.28, 45.64
#49, 14 dpc	Not significant ^3^		8.78	5.67, 19.46	45.38	25.49, 206.47	Not significant ^3^	
#49, 18 dpc	48.81	30.68, 119.22	33.43	21.48, 75.45	22.20	17.5, 30.37	14.27	10.08, 24.48
#50, 18 dpc	46.30	26.23, 197.14	N/D ^2^		4.85	3.03, 12.06	50.39	31.08, 133.03
#106, 18 dpc	Not significant ^3^		57.67	31.06, 403.69	Not significant ^3^		Not significant ^3^	
#919, 18 dpc	45.55	35.09, 64.87	N/D ^2^		3.73	2.69, 6.1	17.81	10.95, 47.72
Medium	129.37	78.03, 378.28	97.58	76.23, 135.54	41.42	34.45, 51.92	51.87	38.85, 78.01

^1^ 95% C.I.: 95% confidence interval. ^2^ N/D: Not determined because the infectious virus was present only at first time point. ^3^ The slope of simple regression model was not significantly different than zero. #: Animal ID number.

## Data Availability

Raw data and the virus titer calculations are available upon request.

## Supplementary Materials

The following supporting information can be downloaded at: https://www.mdpi.com/article/10.3390/v15030761/s1, Table S1: Comparison of consensus amino acid sequences among virus stocks used in this study.
