# Supplementary material for: Stability of SARS-CoV-2 in Biological Fluids of Animals
Source: Viruses. 2023 Mar 16;15(3):761. doi: 10.3390/v15030761 (PMC10058514; doi:10.3390/v15030761)
Supplement: Supplementary file 1 [file viruses-15-00761-s001.zip › viruses-2242538-supplementary.pdf]

Supplementary Table 1. Comparison of consensus amino acid sequences among virus stocks used in this study.

Viral protein	Amino acid position	WA-1 lineage A [1, 2]	Alpha VOC B.1.1.7 [2]	Delta VOC B.1.617.2 [2]	Omicron VOC B.1.1.529 [2]
Spike	19	T	T	R	T
	67	A	A	A	V
	69–70	HV	Deletion	HV	Deletion
	95	T	T	T	I
	142	G	G	D	D
	143–144	VY	VY	VY	Deletion
	145	Y	Deletion	Y	Deletion
	156	E	E	G	E
	157–158	FR	FR	Deletion	FR
	211	N	N	N	Deletion
	212	L	L	L	I
	214				EPE (Insertion)
	242–244	LAL	LAL	LAL	LAL
	339	G	G	G	D
	371	S	S	S	L
	373	S	S	S	P
	375	S	S	S	F
	417	K	K	K	N
	440	N	N	N	K
	446	G	G	G	S
	452	L	L	R	L
	477–478	ST	ST	SK	NK
	484	E	E	E	A
	493	Q	Q	Q	R
	496	G	G	G	S
	498	Q	Q	Q	R
	501	N	Y	N	Y
	505	Y	Y	Y	H
	547	T	T	T	K
	570	A	D	A	A
	614	D	G	G	G
	655	H	H	H	Y
	679	N	N	N	K
	681	P	H	R	H
	701	A	A	A	V
	716	T	I	T	T
	764	N	N	N	K
	771	A	A	S	A
	796	D	D	D	Y
	856	N	N	N	K
	950	D	D	N	D
	954	Q	Q	Q	H
	969	N	N	N	K
	981	L	L	L	F
	982	S	A	S	S
	1118	D	H	D	D
Envelope	9	T	T	T	I
Matrix	3	D	D	D	G

	19	Q	Q	Q	E
	63	A	A	A	T
	82	I	I	T	I
Nucleocapsid	3	D	L	D	D
	13	P	P	P	L
	31–33	ERS	ERS	ERS	Deletion
	63	D	D	G	D
	139	L	L	F	L
	203–204	RG	KR	MG	KR
	235	S	F	S	S
	377	D	D	Y	D
	385	R	R	K	R
NSP2	129	P	P	L	P
NSP3	38	K	K	K	R
	183	T	I	T	T
	822	P	P	L	P
	890	A	D	A	A
	1069	V	V	V	I
	1265	S	S	S	Deletion
	1266	L	L	L	I
	1274	H	H	Y	H
	1305	A	V	A	A
	1412	I	T	I	I
	1892	A	A	A	T
NSP4	446	A	A	V	A
	492	T	T	T	I
NSP5	132	P	P	P	H
NSP6	105	L	L	L	Deletion
	106	S	Deletion	S	Deletion
	107	G	Deletion	G	Deletion
	108	F	Deletion	F	F
	149	V	V	A	V
	189	I	I	I	V
NSP12	323	P	L	L	L
	671	G	G	S	G
NSP13	77	P	P	L	P
	460	K	R	K	K
NSP14	42	I	I	I	V
	347	E	G	E	E
NSP15	234	H	H	Y	H
ORF3a	26	S	S	L	S
	57	Q	Q	Q	Q
	131	W	W	W	W
	171	S	S	S	S
ORF7a	82	V	V	A	V
	116	L	L	F	L
	120	T	T	I	T
ORF8	27	Q	Stop codon	Q	Q
	84	S	Truncated; L	L	L
	119–120	DF	Truncated; DF	Deletion	DF

[1] Two different virus stocks of the WA-1 strain were used in this study. One WA-1 virus stock was grown in Vero E6 cells and was used to determine the SARS-CoV-2 stability in biological fluids derived from

cats, sheep, and white-tailed deer (WTD). The Vero E6 cell derived virus stock contained a KLRS insertion (between amino acid positions 215-216 of the reference sequence) in the spike glycoprotein which was found at a frequency of 44%. The other WA-1 stock virus was grown in Vero-TMPRSS2 cells for side-by-side comparisons with SARS-CoV-2 VOCs in 10% WTD fecal suspension. The Vero-TMPRSS2 cells derived virus stock had a synonymous mutation at position 1912 (C/T) in the nsp2 region.

[2] Between 50 to 120 non-coding sequences of the 5’ and 3’ termini were not determined.
